# Dogs: A Man's Best Friend or a Deadly Beast—A Discussion on *Capnocytophaga canimorsus*

**DOI:** 10.1155/2022/7228214

**Published:** 2022-04-22

**Authors:** Swathi Muttana, Christopher Solowiej Singh, Sabina Dhami, Spyridon Ntelis, Dana Dhami, Miriam Michael

**Affiliations:** ^1^Internal Medicine, American University of Antigua College of Medicine, New York, USA; ^2^Internal Medicine, University of Maryland Medical Center Midtown Campus, Baltimore, USA

## Abstract

*Capnocytophaga canimorsus* is a catalase-positive and oxidase-positive gram-negative bacillus commonly found in dog saliva that is a rare cause of infection in immunocompromised individuals. We report the case of a 70-year-old woman with Waldenström macroglobulinemia treated with ibrutinib and a history of bilateral shoulder arthroplasty and bilateral knee arthroplasty who reported a 1-year history of multi-joint pain and swelling. The patient resides with two pet dogs that often scratch and bite, penetrating the skin, and on culture was found to have *Capnocytophaga canimorsus.*

## 1. Introduction

Joint arthroplasty is a commonly performed procedure often freeing patients from the burden of chronic pain and immobility, improving their overall quality of life. While most procedures are successful, periprosthetic joint infection (PJI) is a dreaded post-surgical complication with the majority of PJIs caused by gram-positive pathogens including *Staphylococci* and *Streptococci* species. These highly virulent organisms typically cause early-onset symptoms, allowing for easy identification of PJI and successful intervention. While *Staphylococci* and *Streptococci* can cause late-onset PJI as well, it is important to keep less common organisms on the differential to ensure adequate treatment [1]. One lesser-known cause of periprosthetic PJI is *Capnocytophaga canimorsus*, a catalase and oxidase-positive, gram-negative staining, facultative anaerobe, transmitted to humans via dog saliva. Here, we present a rare case of *Capnocytophaga canimorsus* PJI in a 70-year-old female, presenting with vague, insidious knee and shoulder pain.

## 2. Case History

The patient is a 70-year-old female with a history of Waldenström's macroglobulinemia on ibrutinib, and a one-year history of bilateral knee and bilateral shoulder pain and swelling. She underwent bilateral total knee arthroplasty (2016) and bilateral shoulder arthroplasty (2018, 2019). One month before the patient's admission, she was seen outpatient by her orthopedic surgeon, in which he aspirated bilateral shoulders and bilateral knees (Table 1). The patient's right shoulder aspiration showed 14,750 white blood cells (WBCs) (95% polymorphonuclear leukocytes (PMNs)), and her left shoulder aspiration revealed 4,356 WBCs (99% PMNs). Her right knee aspiration revealed 31,830 WBCs (86.3% PMNs) with alpha-defensin and staphylococcus positivity, and her left knee aspiration showed 8.002 WBCs (85.7% PMNs) with alpha-defensin and staphylococcus positivity.

The patient was admitted due to concerns of multi-joint periprosthetic joint infection, and underwent aspiration of bilateral shoulders and explant of bilateral knees with antibiotic spacer placement. The patient was initially treated for the assumption of infection secondary to *Staphylococcus* with vancomycin 1.25 g IV every 24 hours. The patient was discharged with prophylactic treatment using daptomycin 500 mg PO daily for 6 weeks.

Several weeks after the patient's discharge from hospital admission, synovasure was positive in her right shoulder for *Capnocytophaga canimorsus* growth in an anaerobic bottle, via 16S ribosomal RNA PCR analysis. The patient confirmed that she resides with two pet dogs that often scratch and bite, penetrating the skin, which is consistent with the growth of *Capnocytophaga*. Given that the patient had underlying immunosuppression secondary to Waldenström's macroglobulinemia on ibrutinib, along with multiple joint surgeries, an aggressive antibiotic treatment regimen was indicated. In addition to prophylaxis of daptomycin 500 mg PO daily for 6 weeks, the patient was started on ertapenem 1 g IV daily for 6 weeks. Revision arthroplasty of bilateral knees and right shoulder is scheduled after the completion of the antibiotic treatment.

## 3. Discussion

### 3.1. *Capnocytophaga canimorsus*

*Capnocytophaga* species are considered slow-growing, capnophilic (carbon dioxide loving), facultative, anaerobes. They are gram-negative staining with a long, thin fusiform appearance. As a species, *Capnocytophaga* can be subdivided into two groups, those inhabiting the human oral cavity and those inhabiting the dog oral cavity. *Capnocytophaga canimorsus* is found specifically in the oral cavity of dogs and cats; therefore, many infections occur in patients with a history of dog bites, scratches, and general exposure [2].

Up to 54% of *C. canimorsus* infections occur due to animal bites and up to 27% of infections are solely due to exposure to animals. Scratches alone cause roughly 8.5% of infections. Though infections commonly occur 2-3 days after animal contact, prior case reports have noted presentation up to 2 years later [3, 4]. Depending on the patient, it may be difficult to elicit a specific history of animal bites or scratches as it may be an insignificant detail to them [3].

Documented manifestations of *C. canimorsus* infections include bacteremia, meningitis, infective endocarditis, sepsis, septic shock, multiorgan failure, and disseminated intravascular coagulation with purpura fulminans [5, 6]. Much of the literature suggests that infections occur most often in patients with risk factors including immunosuppression, asplenia, alcoholism, hematological malignancy, and cirrhosis [3]. However, a recent 10-year retrospective review by Chesdachai et al. noted that only half of the patients with *C. canimorsus* bacteremia possessed these risk factors, suggesting that it is not only an infection of the immunocompromised [7].

*C. canimorsus* elicits its virulence through a variety of mechanisms. As a catalase-positive organism, it can survive in phagocytes by breaking down H2O2. It possesses endotoxin (LPS-lipopolysaccharide) and sialidase, which allow it to evade the immune system by blocking the release of nitric oxide by macrophages and decreasing its interaction with the TLR-4 receptor of the host species [8]. The lipopolysaccharide capsule makes it resistant to both complement killing and phagocytosis by human PMNs. Another important virulence factor is the process in which *C. canimorsus* can harvest iron from transferrin molecules through its PUL3 gene and cleave N-glycoproteins from host cell membranes and proteins such as IgG [9].

While over 100 capsular serovars exist among the species, it has been determined that most human infections are caused by the higher virulence of serovars A, B, and C. However, since these three serovars are a very low percentage of dog strains isolated, it demonstrates why there is a low incidence of disease caused by *C. canimorsus* despite it inhabiting the oral flora of many dogs [10].

Identification of *C. canimorsus* can be difficult given its specific requirements. It grows best on blood or chocolate agar in 10% CO2 while incubated at 37 degrees Celsius for around 5-7 days. Once visible, colonies are smooth in appearance with “finger-like projections,” non-hemolytic, catalase-positive, and oxidase-positive [11]. Due to the slow and variable growth of this organism, methods such as 16S ribosomal RNA PCR have been utilized in several case reports to rapidly identify *C. canimorsus* [4, 12].

Beta-lactamase production has been documented among the *Capnocytophaga* genus in previous literature; therefore, treatment by beta-lactam + beta-lactamase inhibitor (e.g., ampicillin-sulbactam), 3^rd^ or 4^th^ generation cephalosporins, or carbapenems should be used. If susceptibility testing indicates other antibiotics would be ideal, treatment can be adjusted accordingly [7].

### 3.2. Periprosthetic Joint Infection

Periprosthetic joint infection (PJI) is both feared and devastating complication following arthroplasty. This complication is feared due to its association with prolonged hospital admission, additional surgeries, and functional incapacitation [1], which places a financial burden on both the patient and hospital. It is important to identify and treat PJIs because additional-site PJIs in patients with multiple prosthetic joints increase morbidity and risk for the requirement of additional procedures [2]. PJIs are most commonly caused by *Staphylococcus aureus* and *Staphyloccocus epidemics* [3]; however, many additional causes may be overlooked in this setting—*Capnocytophaga canimorsus*, for example.

Our literature review has indicated five reported cases of *Capnocytophaga canimorsus* infecting periprosthetic joints.

Similar to our case, the first case report included a dog-owning patient with underlying risk factors. A case report by Larson et al. described a 59-year-old male dog owner with underlying risk factors similar to the case we presented above. Staged bilateral resection arthroplasties with antibiotic spacer were performed alongside a 6-week course of IV ertapenem and 1 year after the presentation the patient reported doing well with no major symptoms [4].

Other case reports detail patients without immunocompromising factors, such as an 83-year-old male with periprosthetic knee infection successfully treated with long-term antibiotics keeping his prosthetic joint [13]. A 54-year-old male presenting with a year-long right knee pain that started two years after total knee arthroplasty was successfully treated with prosthesis explantation, antibiotic spacer placement, a 4-week intravenous cefuroxime course followed by 2 weeks of oral ciprofloxacin and revision total knee arthroplasty [14]. A 66-year-old female presenting with a 4-month history of groin and hip pain, found to have a prosthetic hip joint infection, underwent a 2-stage revision right total hip arthroplasty with antibiotic spacer placement and reported a pain free hip 15 months later [12]. Finally, a 58-year-old female presenting with insidious left hip pain several weeks after a dog bite, found to have PJI, initially had an explant with irrigation and debridement, placement of antibiotic spacer, and ceftriaxone for 6 weeks. Unfortunately, the patient died of MI before replant surgery could be performed; however, down-trending inflammatory markers noted after antibiotic administration suggest successful treatment as well [15].

### 3.3. Treatment

The patient was initially treated inpatient for her multi-periprosthetic joint infection using vancomycin 1.25 g IV every 24 hours. Upon discharge, the patient was given daptomycin 500 mg PO daily for 6 weeks as prophylactic treatment. Once the 16 s ribosomal RNA PCR analysis indicated growth of *Capnocytophaga canimorsus* in her right shoulder, the patient was aggressively treated using ertapenem 1 g IV daily for 6 weeks, in addition to the continuation of her prophylactic daptomycin treatment.

## Figures and Tables

**Table 1:** Joint aspiration.

	WBC count	% of PMNs in WBC count	Staphylococcus positivity	Alpha-Defensin positivity
Right shoulder	14,750	95	NR	NR
Left shoulder	4,356	99	NR	NR
Right knee	31,830	86.3	+	+
Left knee	8,002	85.7	+	+

WBC: white blood cell; PMNs: polymorphonuclear leukocytes; NR: not reported.
